# Comparative Evaluation of Canal Transport and Centralization Between ProTaper Next and XP-endo Shaper Systems Using CBCT Analysis: An In Vitro Study

**DOI:** 10.1155/ijod/7245596

**Published:** 2025-01-04

**Authors:** Hamed Karkehabadi, Abbas Shokri, Negar Banitalebi, Roshanak Abbasi

**Affiliations:** ^1^Department of Endodontics, Dental Research Center, Avicenna Institute of Clinical Sciences, Avicenna Health Research Institute, Hamadan University of Medical Sciences, Hamadan, Iran; ^2^Dental Implants Research Center, Department of Oral and Maxillofacial Radiology, Faculty of Dentistry, Hamadan University of Medical Sciences, Hamadan, Iran; ^3^Department of Endodontics, School of Dentistry, Lorestan University of Medical Sciences, Khorramabad, Iran

**Keywords:** cone beam computed tomography, endodontics, root canal preparation, root canal therapy

## Abstract

**Aim:** This study compared the apical transportation and centering ratio of ProTaper Next (PTN) and XP-endo Shaper (XPS) nickel titanium (NiTi) rotary files in curved root canals using cone beam computed tomography (CBCT).

**Methodology:** The current in vitro study involved the mesiobuccal canals of mesial roots in 44 extracted mandibular first molars that exhibited apical curvature ranging from 10° to 30°. Two experimental groups were randomly formed from the teeth (*n* = 22) and subjected to instrumentation with PTN and XPS. CBCT scans were performed before and after instrumentation on the teeth, and the apical transport and centering ratio were calculated at 3, 4, and 5 mm from the apex. Group comparisons were conducted using an independent *t*-test with a significance level set at alpha = 0.05.

**Results:** Comparisons within groups did not reveal any statistically significant differences in the magnitude of canal transportation in the buccolingual (BL) and mesiodistal (MD) directions at any level from the apex, neither in the XPS group nor in the PTN group (*p* > 0.05). Canal transportation in both BL and MD directions was significantly greater in PTN than in XPS (*p* < 0.05). The centering ratio in the MD and BL directions was the same at 3, 4, and 5 mm from the apex in the PTN (*p* > 0.05) and also in the XPS (*p* > 0.05) groups. The centering ratio was significantly higher in XPS than in PTN (*p* < 0.05) except at 5 mm from the apex in the MD direction and 4 and 5 mm from the apex in the BL direction (*p* > 0.05).

**Conclusions:** Both buccolingually and mesiodistally, PTN led to greater apical transport than XPS and also showed a lower centering ratio.

## 1. Introduction

Root canal treatment is performed with the aim of cleaning, shaping, disinfecting, and obturating the root canal system using a combination of mechanical instruments (endodontic files) and chemical irrigants. In this process, it is essential not to disrupt the natural structure of the root canal system during treatment. Otherwise, cleaning the root canals and shaping curved root canals can lead to apical transportation [1, 2]. Apical transportation occurs when greater amounts of dentin are removed from the external walls of the apical part of the canal curvature and the internal walls of the coronal part of the canal curvature [3, 4]. The transportation of canals can lead to weaker roots, compromised disinfection, and a greater chance of canal obstruction. Canal transportation in the apical third of the root may compromise the integrity of the root canal system, resulting in residual debris and microorganisms that are difficult to remove [5].

Procedural errors can occur during root canal preparation when using hand or rotary instruments [6].

Since their introduction in 1990, nickel titanium (NiTi) rotary files have significantly improved the quality, efficiency, and speed of root canal instrumentation, especially in curved root canals [7]. Rotary instruments are superior to stainless steel hand files in terms of cutting efficiency and flexibility. Due to their superelasticity, these instruments are able to maintain the original conical shape of the canal during instrumentation and reduce the likelihood of canal transportation [8].

Over time, different NiTi rotary systems were introduced to the market with different tip designs, taper, pitch, rake, and helical angle [6].

The introduction of NiTi single-file rotary systems to the endodontic market has made them more accessible and user-friendly for novice operators. XP-endo Shaper (XPS) (XP, FKG Dentaire SA, Switzerland) as an canal conforming rotary system [9, 10] is a novel instrument made from max-wire alloy [11]. This file has a primary taper of 0.01 in the M phase at cool temperature; however, at a temperature of 35°C, according to the A phase of the molecular memory, the taper of the file transforms to 0.04. According to the manufacturer's statement, the XPS file tip incorporates six cutting blades, allowing the instrument to begin shaping the canal following the glide path ISO 15, and gradually amplifies its effectiveness to attain ISO 30 [9]. Intracanal irregularities do not pose a significant challenge to the XPS material, which demonstrates an impressive ability to adapt and exhibits superior resistance to cyclic fatigue [12].

The ProTaper Next (PTN) system, developed by Dentsply Maillefer in Ballaigues, Switzerland, has a unique asymmetric feature that enables only two cutting blades to come into contact with the canal wall while undergoing a continuous rotation. The present system is fabricated using NiTi M-Wire and has superior mechanical characteristics compared to standard NiTi instruments [13].

A current issue is the lack of a universally accepted method for assessing the centering ability of files and their potential to cause transportation [14]. Thus, several techniques have been proposed for this purpose. To assess the shape of the canal, it is helpful to visually evaluate the canal cross-sections at different levels from the apex. However, this method is inadequate to reveal the original canal pathway before instrumentation. Additionally, the Bramante method can also be utilized to accomplish this. This method involves sectioning the canals before instrumentation. The specimens are reassembled and equipped with instruments, after which they are sectioned once more to evaluate the alterations induced by the instrumentation [15]. Radiographic superimposition is another technique that can reveal changes in two dimensions. It can be stated that the technique is both low-cost and simple in its execution. However, the two-dimensional (2D) nature of images imposes certain constraints [16]. High-resolution cone beam computed tomography (CBCT) images may also be used for this purpose. This technique has proven to be highly promising, reproducible, and noninvasive and allows evaluation of root canal anatomy and shape before and after preparation. It allows for a precise three-dimensional (3D) evaluation of root canal changes that can be quantified by software, although it is costly and time-consuming [15, 17].

There is limited research comparing the apical transportation and centering ability between the XPS and PTN systems, particularly using advanced imaging techniques such as CBCT in controlled conditions that simulate the clinical environment. This study aims to address this gap by conducting a comparative analysis of both mesiodistal (MD) and buccolingual (BL) canal transportation, which are critical for preserving root canal anatomy and ensuring treatment success. We hypothesize that the XPS and PTN systems will not differ significantly in performance for these key parameters, and the results of this analysis will either confirm or reject this hypothesis. Additionally, the simulation of clinical conditions, including the maintenance of body temperature during instrumentation, introduces a novel and clinically relevant dimension to the evaluation. By advancing our understanding of these rotary systems, this research is expected to make a significant contribution by diversifying the body of evidence on canal transport phenomena and informing clinical guidelines on the optimal usage of XPS and PTN tools, ultimately improving decision-making and outcomes in endodontic practice.

## 2. Methods

An experimental in vitro study was conducted on 44 extracted mandibular first molars with apical curvature ranging from 10° to 30°. The teeth were obtained from a pool of teeth previously extracted for nonrelated purposes such as orthodontic reasons, hopeless periodontal prognosis, or extensive caries.

The approval for the study was obtained from the Ethics Committee of the Hamadan University of Medical Sciences (reference IR.UMSHA.REC.1402.157). It is important to note that the extracted teeth used in our investigation were obtained for reasons not related to the study, such as caries, periodontal concerns, and other dental health issues. Before the extraction procedure in the Department of Oral and Maxillofacial Surgery, all patients provided their informed consent for their teeth to be used for research purposes. This consent was obtained by filling out a dedicated form, ensuring that the participants were fully informed about the nature of the study. We affirm that all methodologies were strictly conducted in accordance with applicable regulations and laws, and the ethical principles outlined in the Declaration of Helsinki guided our investigators throughout the study.

### 2.1. Sample Size

The sample size determination for this study was grounded in the results of a previous investigation conducted by Madani et al. [18], which assessed canal transportation and centering ability using a similar methodology. To ensure sufficient power for detecting significant differences between the two groups (PTN and XPS systems), a power analysis was conducted prior to the study. Using the findings from Madani et al., we established critical parameters for the power calculation, including an effect size (Cohen's *d*) of 0.8, indicating a large effect based on observed differences in canal transportation between rotary systems, a power of 0.80, and an alpha (*α*) level of 0.05. This resulted in a required sample size of 22 specimens per group, yielding a total sample size of 44 specimens. This carefully calculated sample size justifies our methodological approach and ensures the study is adequately powered to detect clinically relevant differences in canal transportation and centering ability between the two file systems, thus providing confidence in the robustness of our findings.

### 2.2. Eligibility Criteria

The current in vitro study involved the mesiobuccal canals of the mesial roots in 44 extracted mandibular first molars that exhibited apical curvatures ranging from 10° to 30°. The experimental sample encompassed the mesial roots of first mandibular molars exhibiting mesiobuccal curvature of 10°–30°. Exclusionary factors during screening procedures included teeth with root fractures, internal/external resorptive defects, immature apices, S-shaped canal morphologies, inability to negotiate a #10 file to working length, and masking artifacts of metal indicators that interfere with CBCT visualization. The samples were examined at 5× magnification using a ZEISS dental operating microscope to detect microcracks or other defects that would compromise structural integrity prior to canal instrumentation. Teeth showing visible fractures under high magnification were excluded to avoid confounding results. Furthermore, any cases that resulted in file separation were excluded from the analysis to avoid confounding the evaluative data.

### 2.3. Methodology

Specimens were subjected to standardized disinfection by immersion in 5.25% sodium hypochlorite solution. Digital periapical radiographs (60 kVp, 6 mA, and 0.12 s; Minray, Soredex) assessed canal curvature angles based on Schneider's methodology, facilitated by Scanora software for precise caliper measurements [19]. A longitudinal line was marked on the tooth, and a line was drawn from the apical foramen to the point where the canal first deviated from the longitudinal axis. An acute angle was formed as such, which was measured with a caliper to determine the angle of curvature of the tooth. Teeth with 10°–30° curvature (mesiodistally) in their mesial root were selected. Teeth exhibiting 10°–30° mesial curvature (*n* = 22 per group) were assigned to standardized PTN or XPS instrumentation arms.

The establishment of an access cavity in the teeth was performed using a high-speed carbide bur (Dentsply, Maillefer, Ballaigues, Switzerland). The determination of the working length involved measuring the distance between the occlusal reference point and a location 1 mm shorter than the length of a K-file #10 (Dentsply, Maillefer, Ballaigues Switzerland). Establishing the working length was based on direct visualization of a #10 file at the apex, subtracting 1 mm to set the instrumentation endpoint [20].

The acrylic embedding introduced radiopaque orientation markers to facilitate imaging analyses.

CBCT imaging before instrumentation (CS9600, Carestream) involved high-resolution parameters (60 kVp, 10 mA, 12 s, and 90-μm voxels). The DICOM files generated established the comparative baseline anatomy. A standardized #15 K file enhanced glide path patency prior to rotary shaping procedures in compliance with manufacturer guidelines for speed and torque.

In group A (*n* = 22), the mesiobuccal canal of the mesial roots was instrumented with PTN (Dentsply Maillefer, Ballaigues, Switzerland). The instrumentation of all canals was carried out by an identical operator. The endomotor (X-Smart Plus motor) was the instrument of choice for the operator to instrument all canals with a torque of 2 N/cm and a speed of 300 rpm. *X*1 was first used, and then *X*1 and *X*2 were used to the working length. After the use of each file, the root canals were irrigated with 2 mL of a 2.5% sodium hypochlorite and saline solution.

The preparation of the root canal in group B involved XPS (Dentaire, La Chaux-de-Fonds, Switzerland). To replicate body temperature, teeth were immersed in water at 37 ± 1°C as measured by a thermometer during the instrumentation process of instrumentation [21]. The torque and speed of XPS were 1 N/cm and 800 rpm, respectively. Upon insertion into the canal, the file tip was activated with rotation. Having reached the working length, the up-and-down motion was repeated five times. Recapitulation was performed using a #15 file after the usage of each file, and the canal was rinsed with 2 mL of sodium hypochlorite 2.5% and saline [9, 22]. The files were intended for the preparation of two canals each and no more.

Subsequently, CBCT was conducted on teeth using exposure parameters identical to those from baseline. OnDemand 3D Dental software (CyberMed, Seoul, South Korea) was utilized to determine canal transport and centering ratio.

**Figure 1:**
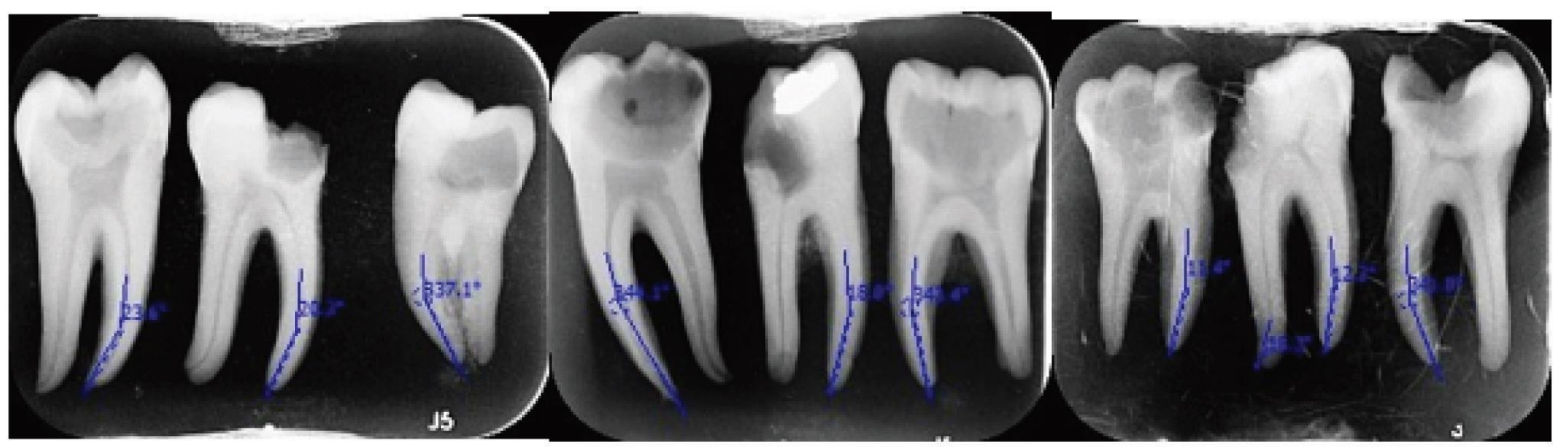
Measurement of dentin thickness in the sagittal plane involved drawing a line along the root curvature to ensure sections were entirely perpendicular to the tooth's longitudinal axis.

To measure the thickness of dentin in the sagittal plane (Figure 1), a line was drawn along the root curvature to ensure that the sections are completely perpendicular to the longitudinal axis of the tooth. Next, in the axial sections, the thickness of the dentin was measured 3, 4, and 5 mm from the apex on the mesial, distal, buccal, and lingual surfaces with a cut thickness of 0.5 mm and a cut interval of 0.5 mm using the ruler and sectioning feature of the OnDemand 3D Dental software program (Cybermed, Seoul, South Korea) and reported in millimeters (mm). Due to variations in the anatomy of the canal curvatures and for the purpose of standardization, the root canals were evaluated at three levels [23]. The same was also performed on postinstrumentation CBCT images.

**Figure 2:**
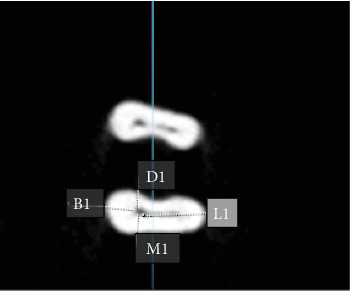
Measurement of dentin thickness on axial cone beam computed tomography (CBCT) sections in the mesial, distal, buccal, and lingual surfaces of the mesiobuccal root.

**Figure 3:**
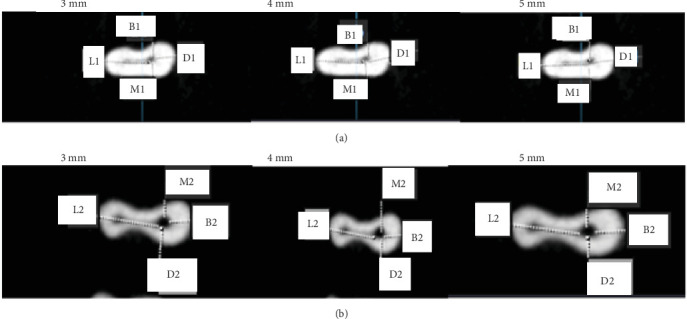
Cone beam computed tomography (CBCT) images before (A) and after (B) instrumentation of the mesiobuccal canal by ProTaper Next (PTN) at 3, 4, and 5 mm from the apex.

**Figure 4:**
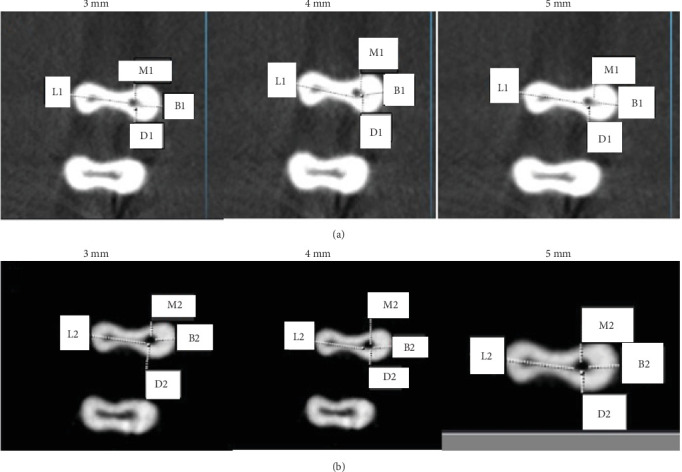
Cone beam computed tomography (CBCT) images before (A) and after (B) instrumentation of the mesiobuccal canal by XP-endo Shaper (XPS) at 3, 4, and 5 mm from the apex.

The canal transport and centering ratio was calculated in the MD and BL dimensions using the following formula in three cross-sections 3, 4, and 5 mm from the root apex using the following formula [24] (Figures 2–4):

  $$\begin{array}{c} \text{MD transportation}=\left(M1-M2\right)-\left(D1-D2\right), \end{array}$$

  $$\begin{array}{c} \text{BL transportation}=\left(B1-B2\right)-\left(L1-L2\right), \end{array}$$

where M1, M2, D1, and D2 represent the minimum distance from canal border to the respective root border before and after instrumentation. Similarly, B1, B2, L1, and L2 denote minimum distances in buccal and lingual planes. A value of 0 signifies no transport.

The centering ratio was quantified in both MD and BL planes with the following:

  $$\begin{array}{c} \text{MD ratio}=X/Y, \end{array}$$

  $$\begin{array}{c} \text{BL ratio}=X/Y. \end{array}$$

Here *X* is the lowest value and *Y* the highest value of the two ratios calculated from the minimum canal to root border distances before and after instrumentation in each plane. A ratio value of 1 indicates a perfect centered preparation.

The measurements were made by a senior dental student who was experienced using OnDemand 3D Dental software and an experienced oral and maxillofacial radiologist.

To assess the intraobserver agreement, both observers re-evaluated all CBCT images and repeated all the measurements after 1 month. The interobserver agreement was also calculated. The distribution of the sample into groups was concealed from both observers. To get the finest visual effects, they were both given the freedom to change the images' brightness, contrast, and magnification.

### 2.4. Statistical Analysis

Quantitative data were processed using SPSS software version 24.0 (SPSS Inc., Illinois, USA). The descriptive statistics included means and standard deviations for both study groups. Normality was confirmed using the Kolmogorov–Smirnov test to validate the appropriateness of the parametric analysis. Comparison of transportation and centering metrics between the XPS and PTN systems was performed using independent sample *t* tests, while intragroup comparisons relied on Wilcoxon signed rank assessments. Statistical significance was defined with an alpha of 0.05 for all analytical procedures.

## 3. Results

Reliability analysis revealed robust intraobserver agreement (91%) and interobserver agreement (87%), validating transportation and centering ratio methodology.

### 3.1. Canal Transportation

According to the data presented in Table 1, comparative analysis of canal transport in MD and BL dimensions induced by the PTN and XPS rotary systems revealed the following key findings.

Intragroup comparisons did not show significant differences (*p* > 0.05) in the extent of deviation from original anatomy between the axial levels of 3, 4, and 5 mm for the PTN or XPS instruments, indicating consistency of the transportation behavior within each system.

However, the intergroup analysis showed significantly greater transportation for PTN files compared to XPS rotaries (*p* < 0.05) at critical apical distances of 3, 4, and 5 mm in both BL and MD orientations.

In aggregate, these results decisively reveal a propensity for the adjustable taper XPS system to better preserve native canal trajectory and minimize aberrant apical transportation compared to the shape memory PTN NiTi rotary instrumentation across clinically relevant regions of interest.

### 3.2. Centering Ratio

Regarding the critical centering ability parameter as presented in Table 2, comparative analysis between the PTN and XPS rotary systems demonstrated the following key trends.

The intragroup evaluation did not show significant differences in MD or BL centering ratios (*p* > 0.05) between the axial planes of 3, 4, and 5 mm for the PTN or XPS instruments, indicating consistency within each system.

However, intergroup comparisons revealed decisively greater centering precision for the XPS rotary technique relative to the PTN approach at all apical levels, both mesiodistically and buccolingually. In particular, in the MD orientation, XPS achieved significantly enhanced centralization at 3 mm (*p* < 0.05) and 4 mm (*p* < 0.05) from the apex. Additionally, the superiority of the XPS system over PTN instruments was more pronounced buccolingually near the apex at 3 mm (*p* < 0.05).

In total, these central data offer corroborative quantitative evidence that the novel adjustable taper XPS design better maintains the original canal trajectory compared to traditional rotaries, minimizing aberrant transportation across regions of interest critical for endodontic success.

## 4. Discussion

Numerous studies have evaluated canal transportation and centering ability between PTN and XPS systems under various conditions, such as large canals, curved canals, and even the isthmus region. However, the current study offers a unique contribution by focusing on a highly controlled in vitro environment using CBCT imaging. What sets this study apart is the simultaneous evaluation of both MD and BL dimensions of canal transportation, providing a more comprehensive analysis compared to prior research. Additionally, the simulation of clinical conditions through maintaining body temperature (37°C) during instrumentation introduces an innovative aspect that more closely mirrors the intraoral environment. These factors enhance the clinical relevance of the findings and offer new insights into the comparative performance of PTN and XPS systems in curved canals.

The current study compared the performance of two NiTi rotary systems, the novel XPS and the established PTN, assessing key parameters of apical transportation and centering ratio. We hypothesized that there would be no significant differences between XPS and PTN for these metrics. However, our findings showed that XPS demonstrated significantly less canal transportation and improved centering ability compared to PTN, leading us to reject our null hypothesis. Specifically, at clinically relevant distances of 3, 4, and 5 mm from the root apex, transportation increased markedly with the PTN system compared to XPS in both BL and MD dimensions. Kabil et al. [25] reported similar results when comparing the transportation of rotary file systems, including PTN, Reciproc Blue, Reciproc, XPS, and TruNatomy. Morales et al. [26] compared iRace, Reciproc Blue, WaveOne Gold, and XPS and reported that XPS caused lower canal transportation and had a higher centering ratio than other file systems, which was in line with the present results. Karkehabadi et al. [27] compared canal transportation and centering ability of XPS and universal ProTaper and reported a lower transportation and a higher centering ratio of XPS, which was in line with the present results. Öztürk, Ateş, and Fişekçioğlu [28] compared the transportation of XPS and PTN in large root canals and reported results similar to the present findings.

The higher centering ratio and the lower canal transport in the use of XPS can be due to the design and manufacturing process of this system [29]. XPS is made of heat treated wire, which has optimal superelasticity and increases the flexibility of the file, preserves the integrity of the canal, and minimizes canal transportation [30]. The transformation of the alloy is also another reason that may explain its superior performance. XPS has triangular booster tip cross-sectional design, while the cross-sectional design of PTN is triangular and convex. Despite its benefit in improving the flexibility of the file, the triangular cross-section also amplifies its cutting efficiency, which can lead to the undesirable and excessive elimination of dentin from the canal walls, eventually causing canal transportation [31]. Furthermore, the type of movement of the two file systems may explain their different behaviors in terms of deviation from the original canal path. Although both XPS and PTN have continuous rotational movement, PTN has a centric, while XPS has eccentric and semicircular rotation [32].

Greater apical transportation in the PTN group, in comparison with XPS, may be attributed to the type of PTN alloy. It is fabricated from a conventional NiTi alloy, which tends to straighten up in the canal curvature and return to its original shape because of its shape-memory property. This property can exert unbalanced lateral forces to the canal walls, resulting in excessive removal of the root canal wall structure (from the external wall) and lead to inadequate cleaning of the internal wall of the canal curvature. However, the XPS file has a spiral form and is fabricated from a specific max-wire NiTi alloy subjected to alloy treatment and changes shape following thermal alterations [33]. The XPS file has 0.01 taper; however, when placed in an oral environment, its taper increases to 0.04. Its magnitude of expansion depends on the anatomy of the canal, which decreases canal transportation [29]. An earlier study demonstrated that the asymmetrical structure of the ProTaper file with 0.06 taper allowed it to remove a similar amount of dentin as an instrument with 0.08 taper [23].

In the current research, the magnitude of canal transport in the BL direction was highest at 3 mm from the apex, probably due to maximum curvature. Canal curvature can lead to an unequal stress distribution in the file and increase the occurrence of procedural errors.

In the present study, MD transport was significantly the lowest at 3 mm from the apex in the XPS group, which is a great advantage of this system considering the presence of a danger zone in the mesial root toward the furcation (distal root surface).

In the present study, PTN showed a significantly lower centering ratio, which was in line with the results of Al Mutairi, Badr, and Kataya [34], which compared PTN with WaveOne Gold, ProTaper Gold, and WaveOne files. Also, Öztürk, Ateş, and Fişekçioğlu [28] reported superior centering ratio of XPS compared to PTN; however, they evaluated straight canals and attributed this finding to the multifile nature of PTN and its different taper. Moreover, lower centering ratio of PTN can be attributed to the variable taper of the cutting blade along its length (unlike XPS which has a constant taper along the file). It has been established that instruments with constant apical inclination demonstrate superior centering ability compared to those with variable and progressive taper along the blade [35], because progressive inclination increases stiffness and decreases file flexibility and can lead to canal transportation [36]. This statement was confirmed by Saleh et al. [37] as well.

It should be mentioned that XPS files have higher resistance and cyclic fatigue flexibility than PTN, and some other files due to their maximum wire alloy explain their superior performance in curved root canals [38]. Nathani et al. [39] compared PTN with self-adjusting file system and reported a lower transportation and higher centering ratio of self-adjusting file compared to PTN. Their results were different from those of the present findings, probably because of the different types of alloys used for the manufacturing of the files. Pansheriya et al. [40] compared transportation and centering ratio of the PTN and V-Taper files and reported that the PTN caused no apical transportation and had an excellent centering ratio. Their results were in contrast to the present findings, which may be due to the fact that V-Taper is made of stainless steel, which has lower flexibility than NiTi, which is used for the fabrication of PTN.

Apical transportation >0.3 mm can compromise the apical seal and endanger the prognosis of treatment [41]. Despite the presence of a significant difference in apical transport between the two groups in the present study, the transportation range was 0.03–0.19 mm, which does not compromise the apical seal. Both rotary systems caused minimal apical transportation and successfully maintained the apical seal.

In comparison of our study with the study of Nayez et al. [42], notable distinctions arise in the comprehensive analysis of root canal instrumentation results. It should be noted that, despite the methodological differences outlined, our results align in a consistent manner. While previously published work focused solely on the MD aspect when evaluating the centering ratio and transportation, our investigation delves further by scrutinizing both MD and BL aspects. This expanded approach provides a more complete understanding of the effects of root canal shaping. Furthermore, our study introduced a novel dimension by simulating the oral temperature of 37°C before instrumentation, reflecting the clinical environment. This temperature simulation contributes to the contextual relevance of our findings, acknowledging the dynamic conditions encountered during clinical procedures. These nuanced methodological differences enhance the depth and applicability of our study, distinguishing it and contributing valuable information to the existing body of literature. When looking at our study compared to Yusra and Shukri [43], notable differences emerge in both experimental design and analysis of root canal instrumentation results. We conducted our investigation using the first extracted human mandibular teeth, a choice that adds clinical relevance by involving authentic biological specimens. In contrast, the published article relied on ready-made simulated curved canals crafted from clear polyester resin. While this approach standardized the experimental setup, it potentially lacked the intricacies inherent in the natural anatomy of teeth. Additionally, our study is notable for examining both MD and BL aspects during the evaluation of centering ratio and transportation, providing a more comprehensive understanding of the effects of root canal shaping. On the contrary, the published article focused on the inner aspect in the middle parts of the canal and the outer aspect of the curve at the apex, offering a different perspective on the morphology of the root canal.

Although extensive efforts were made to ensure a clinically realistic model, standardization of certain variables remains a challenge. Natural dentin thickness varies intrinsically between samples, which can impact the dynamics of heat dissipation along the canal surface during file manipulation [44]. Our reliance on extracted teeth inevitably introduces heterogeneity arising from the underlying health conditions that warrant removal (e.g., severe periodontal or carious pathology and orthodontic realignment), further influencing the integrity of the dentin inherent dentin integrity in complex ways [44]. Importantly, the innovative XPS system undergoes a phase transition upon exposure to body temperature, which affects file behavior. Thus, strict temperature regulation is imperative. In this study, all samples were stored at 37°C after extraction to reasonably simulate the intraoral condition. In fact, temperature affects the mechanical performance of all NiTi formulations [45]. Although extrapolating laboratory results to in vivo scenarios must be done judiciously, our experimental protocol addressed key physiological parameters through regulated thermal storage. Additional clinical studies are warranted to complement these initial simulated findings. Multifactorial approaches, which include usage across various tooth types and natural dentin states, will further delineate the promising benefits of the XPS system over traditional NiTi rotaries.

Contemporary studies endorse CBCT, computed tomography, and microcomputed tomography (microCT) as more effective than traditional radiographs in evaluating canal shaping and instrumentation. Although microCT offers high-resolution images for precise quantification, its widespread adoption faces challenges such as high equipment costs, prolonged scan/reconstruction times, and a high radiation dose, limiting its in vivo applications [28, 46–48]. This study utilized a specific endodontic CBCT protocol, capturing scans at a 75-μm voxel size, which struck a balance between resolution and field of view. This approach aimed to reliably discern canal anatomy while minimizing confounders. Further refinements in imaging and analytic techniques are warranted. Additional curved canals, system comparisons, and in vivo validations should be analyzed. Quantifying dentin removal and shaping could elucidate the reduced apical transport with XPS, attributable to its adjustable design. Although controlled testing establishes an important foundation, clinical implementations across expanded samples are essential to confirm XPS advantages in centering and transportation over NiTi rotaries. This preliminary research reveals XPS improvements over PTN. Comprehensive XPS evaluation through multifactor use is needed to cement enhancements. In vitro testing ensures anatomy maintenance under standardized conditions but lacks natural challenges. CBCT may miss ultrafine changes visible by microCT. Generalizability is limited by focusing solely on mandibular molars. Future studies should evaluate performance across tooth types and aberrancies beyond transportation. Long-term randomized controlled trials would provide high-quality evidence on the outcomes. This examined moderately curved canals; further analyses should evaluate more severely curved morphologies. Additional comparisons with novel systems may provide fuller differentiation, taking into account the operator and protocol variables. Both ex vivo and in vivo validation are warranted to substantiate these findings.

## 5. Limitations and Clinical Implications

One of the key limitations of this study lies in its in vitro design, which, while allowing for a controlled and standardized assessment of canal transportation and centering ability, may not fully replicate the complexities of a clinical setting. Extracted teeth are devoid of the natural periapical environment and the physiological conditions present in the oral cavity, such as continuous blood flow, patient movement, and variations in temperature beyond the controlled laboratory setting. Although we attempted to simulate oral temperature during instrumentation, the lack of dynamic conditions, such as those found in live tissues, restricts the generalizability of the findings. Additionally, inherent variability in root canal anatomy, such as differences in dentin thickness and canal curvature, could impact the clinical outcomes when applying these results to a broader population.

Another limitation pertains to the use of CBCT as the primary imaging modality. While CBCT offers 3D insights and has become a valuable tool in evaluating canal shaping, it may lack the precision of microCT, which could reveal more subtle alterations in root canal morphology. The resolution of CBCT may not detect finer details, such as minimal transportation or small microcracks, which could influence long-term treatment outcomes.

The sample size, though statistically powered based on previous studies, represents only a small subset of root canal configurations, focusing primarily on mandibular molars with specific curvatures. This limits the applicability of the results to other tooth types and more complex canal anatomies, such as S-shaped canals or those with extreme curvatures.

Despite these limitations, the findings of this study provide valuable insights into the performance of the PTN and XPS systems in minimizing apical transportation and optimizing centering ability. In clinical practice, the superior centering ability and lower transportation exhibited by the XPS system, particularly in curved canals, suggest that it may be better suited for preserving root canal anatomy in cases with complex curvatures. The XPS system's enhanced flexibility, due to its unique alloy and adaptive shaping, may reduce the risk of procedural errors such as canal straightening or excessive dentin removal, which can compromise the structural integrity of the tooth and reduce the success of endodontic therapy.

The clinical relevance of these results is that the use of XPS could lead to better preservation of dentin, lower incidence of apical transportation, and more predictable shaping outcomes, particularly in cases where maintaining the original canal anatomy is critical. This could improve the prognosis of endodontic treatment by reducing the likelihood of canal obstruction or inadequate disinfection in curved canals. However, further clinical studies, particularly randomized controlled trials, are needed to validate these findings in vivo and across a wider variety of tooth types and root canal configurations.

## 6. Conclusion

PTN had a lower centering ratio and caused greater apical transport in both BL and MD directions compared to XPS.

## Figures and Tables

**Table 1:** Apical transport in mesiodistal and buccolingual directions caused by PTN and XPS.

Direction	Rotary system	3 mm	4 mm	5 mm	Within-group comparison*⁣*^*∗*^
		Mean ± SD	Mean ± SD	Mean ± SD	*p*-Value
Buccolingual	PTN	0.404 ± 0.427	0.354 ± 0.39	0.33 ± 0.337	<0.285
	XPS	0.115 ± 0.102	0.089 ± 0.102	0.076 ± 0.096	<0.994
	*p*-Value	0.002	0.002	0.004	—

Mesiodistal	PTN	0.13 ± 0.17	0.13 ± 0.18	0.166 ± 0.189	<0.657
	XPS	0.04 ± 0.05	0.09 ± 0.11	0.76 ± 0.0942	<0.887
	*p*-Value	<0.001	0.05	0.029	—

Abbreviations: PTN, ProTaper Next; XPS, XP-endo Shaper.

*⁣* ^*∗*^The Wilcoxon test.

**Table 2:** Centering ratio of PTN and XPS in mesiodistal and buccolingual directions.

Direction	Rotary system	3 mm	4 mm	5 mm	Within-group comparison*⁣*^*∗*^
		Mean ± SD	Mean ± SD	Mean ± SD	*p*-Value
Buccolingual	PTN	0.34 ± 0.35	0.292 ± 0.46	0.26 ± 0.44	0.847
	XPS	0.256 ± 0.59	0.297 ± 0.55	0.25 ± 0.53	0.855
	*p*-Value	0.019	0.35	0.28	—

Mesiodistal	PTN	0.213 ± 0.347	0.225 ± 0.377	0.276 ± 0.488	0.593
	XPS	0.276 ± 0.66	0.218 ± 0.54	0.246 ± 0.586	0.501
	*p*-Value	<0.001	<0.029	0.255	—

Abbreviations: PTN, ProTaper Next; XPS, XP-endo Shaper.

*⁣* ^*∗*^The Wilcoxon test.

## Data Availability

Although the datasets generated and analyzed during this investigation are not available to the public, they can be accessed from the corresponding author upon reasonable request.

## Nomenclature

NiTi: Nickel titanium
CBCT: Cone beam computed tomography
PTN: ProTaper Next
XPS: XP-endo Shaper
mm: Millimeter
s: Seconds
microCT: Microcomputed tomography.
